# Case Report: Strangulated transverse colon inguinal hernia complicated by cecal perforation in a patient with sclerosing mesenteritis

**DOI:** 10.3389/fsurg.2026.1851753

**Published:** 2026-05-21

**Authors:** Yu Yuan Chang, Je Ming Hu, Yu Cheng Chiu

**Affiliations:** 1 Division of Urology, Department of Surgery, Tri-Service General Hospital, National Defense Medical University, Taipei, Taiwan; 2 Division of Colorectal Surgery, Department of Surgery, Tri-Service General Hospital, National Defense Medical University, Taipei City, Taiwan; 3 School of Medicine, College of Medicine, National Defense Medical University, Taipei City, Taiwan; 4 Graduate Institute of Medical Sciences, College of Medicine, National Defense Medical University, Taipei City, Taiwan; 5 Department of General Surgery, Tri-Service General Hospital, National Defense Medical University, Taipei City, Taiwan

**Keywords:** case report, inguinal hernia, sclerosing mesenteritis, strangulation ileus, transverse colon

## Abstract

**Background:**

Transverse colon herniation into the inguinal canal is exceptionally rare. The transverse colon, being entirely intraperitoneal and suspended by the transverse mesocolon, is anatomically resistant to inferior displacement. Few cases of transverse colon herniation into inguinal or femoral hernias are documented, most occurring in the presence of giant hernia defects, malignancy, or concurrent omental herniation. We report an unusual case of isolated transverse colon strangulation within a right inguinal hernia in the setting of sclerosing mesenteritis (SM), suggesting a novel pathological mechanism for this rare clinical entity.

**Case presentation:**

A 58-year-old man presented with acute abdominal pain and an irreducible right groin mass. Computed tomography demonstrated the transverse colon herniated into the right inguinal canal, cecal dilatation, and pneumoperitoneum. Emergency laparotomy revealed a necrotic transverse colon, a perforated cecum, and fibrotic mesenteric plaques with mesenteric shortening. The patient underwent right hemicolectomy, protective loop ileostomy, and Bassini herniorrhaphy. Histopathology confirmed mesenteric fibrosis without evidence of malignancy. The postoperative course was uneventful, and ileostomy closure was performed three months later.

**Conclusion:**

We propose that mesenteric shortening secondary to sclerosing mesenteritis displaced the transverse colon inferiorly, facilitating its herniation through a pre-existing inguinal defect. This potential association suggests that mesenteric fibroinflammatory disease may warrant consideration when encountering atypical hernia contents.

## Introduction

Inguinal hernia is one of the most common conditions in general surgery, with an annual incidence of approximately 175 per 100,000 population and a lifetime risk of 27% in men and 3% in women (1). Although most inguinal hernias are reducible, strangulation remains a life-threatening complication, with previously reported cumulative probabilities of 2.8% at 3 months, 4.5% at 2 years, and 8.6% at 5 years (2).

The contents of an inguinal hernia sac can vary. In a surgical series of 94 incarcerated inguinal hernias, the most frequent contents were the small intestine (54%) and omentum (38%), whereas the large bowel was involved in only 6% of cases (3). Large-bowel herniation typically occurs as a sliding hernia involving the sigmoid colon on the left or the cecum on the right; in contrast, transverse colon herniation is exceptionally rare (4). Our literature review identified only 13 previously reported cases (5–17). The predisposing factors described in these cases include traction by co-herniating omentum, giant hernia defects with loss of abdominal domain, and malignancy serving as a propulsive lead point. However, no previous report has implicated sclerosing mesenteritis (SM)—a rare fibroinflammatory disorder of the mesentery that can cause mesenteric shortening and bowel loop tethering—as a predisposing factor (18).

## Case presentation

**Figure 1:**
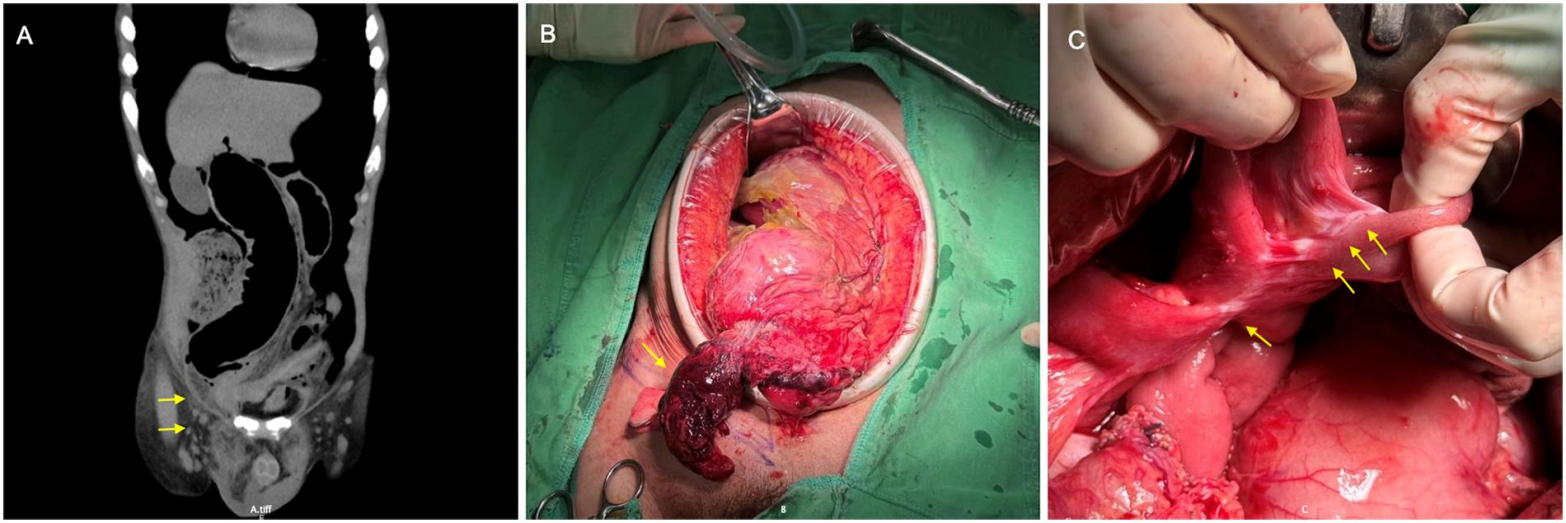
**(A)** A coronal computed tomography scan shows a loop of the transverse colon herniated into the right inguinal canal (yellow arrow), with associated marked dilatation of the ascending colon and cecum; **(B)** An intraoperative view demonstrates the incarcerated transverse colon within the right inguinal hernia sac, with ischemic necrosis and severe cecal dilatation (yellow arrow); **(C)** An intraoperative view reveals multiple fibrotic plaques on the mesentery (yellow arrow), with associated mesenteric shortening and tethering of small-bowel loops consistent with sclerosing mesenteritis.

A 58-year-old man with a history of gout and multiple tophi presented to the emergency department with progressive abdominal pain, nausea, vomiting, and right groin swelling for two days. He reported that the right groin swelling had been present for several months and was previously reducible, but had become irreducible over the past 48 h. The abdominal pain was initially intermittent but became persistent and progressively worsened prior to presentation. On presentation, the patient's vital signs were as follows: temperature 37.1 °C, heart rate 147 beats/min, blood pressure 143/90 mmHg, and respiratory rate 26 breaths/min. Physical examination revealed a tender, irreducible mass in the right groin accompanied by diffuse abdominal tenderness. Laboratory investigations revealed leukocytosis with a left shift, a markedly elevated C-reactive protein level (43.1 mg/dL), lactic acidosis (3.1 mmol/L), and elevated serum creatinine level (1.9 mg/dL). Given the renal impairment, a non-contrast computed tomography scan of the abdomen was obtained, revealing a loop of the transverse colon herniated into the right inguinal canal, with surrounding fat stranding, marked cecal dilatation, pneumoperitoneum, and bilateral hydronephrosis (Figure 1A). An emergency laparotomy demonstrated a necrotic transverse colon within the right inguinal hernia sac and a perforated cecum with severe dilatation of the ascending colon (Figure 1B). Additionally, clustered adhesions between small-bowel loops and multiple whitish mesenteric plaques were noted (Figure 1C). No accompanying omentum was identified within the hernia sac; instead, the omentum remained intra-abdominal and extended into the pelvis. The surgical intervention consisted of a right hemicolectomy, antiperistaltic ileocolostomy, protective loop ileostomy, and Bassini herniorrhaphy. Histopathology revealed transmural necrosis of the colonic segment, while the mesenteric plaques showed fibrotic nodules of mesenteric tissue with dense collagen deposition, fibroblast proliferation, and sparse chronic inflammatory infiltration, without evidence of malignancy. Serological testing excluded IgG4-related disease. In conjunction with the intraoperative findings of mesenteric plaques, mesenteric shortening, and tethering of adjacent small-bowel loops, these findings were considered most consistent with SM within the Emory spectrum, favoring a fibrosis-dominant (retractile mesenteritis) phenotype. Postoperatively, the patient recovered uneventfully, and the obstructive nephropathy resolved. Three months later, the patient underwent successful ileostomy closure with no evidence of hernia recurrence. The key clinical events of the episode of care are summarized in Table 1.

**Table 1:** Clinical timeline of the episode of care.

Time point	Clinical events and key findings
Several months prior to presentation	Right groin swelling.
48 h prior to presentation	The groin mass became irreducible. Abdominal pain became persistent and progressively worsened.
Emergency department presentation	Tachycardia, tachypnea, diffuse abdominal tenderness, and an irreducible tender right groin mass were noted. Laboratory testing showed leukocytosis, markedly elevated C-reactive protein, lactic acidosis, and impaired renal function.
Preoperative imaging	Non-contrast abdominal CT revealed herniation of the transverse colon into the right inguinal canal, marked cecal dilatation, pneumoperitoneum, and bilateral hydronephrosis.
Emergency surgery	Laparotomy demonstrated a necrotic transverse colon within the right inguinal hernia sac, perforated cecum, clustered small-bowel adhesions, multiple whitish mesenteric plaques, and no accompanying omentum within the sac.
Postoperative diagnostic workup	Histopathology revealed transmural colonic necrosis and fibrotic nodules of mesenteric tissue with dense collagen deposition, without evidence of malignancy. Serologic testing excluded IgG4-related disease.
3 months post-surgery	Successful ileostomy closure was performed. No hernia recurrence was observed.

## Discussion

Groin herniation of the transverse colon is exceptionally rare. This rarity is due to the colon's stable anatomical fixation by the transverse mesocolon and its secure attachments at the splenic and hepatic flexures. We conducted a literature search of the PubMed/MEDLINE and Google Scholar for the period 1996–2026 using the terms (“transverse colon”) AND (“inguinal hernia” OR “femoral hernia”). The reference lists of all identified articles were also reviewed for additional relevant cases. Only cases in which the transverse colon was explicitly identified as a component of the hernia sac were included. Our search identified 13 previously reported cases across inguinal hernia or femoral hernia, in addition to the present case (Table 2).

**Table 2:** Comparative summary of previously reported cases of transverse colon groin herniation.

No.	Author (Year)	Age/Sex	Hernia Type	Side	Sac Contents	Complications	Proposed Mechanism	Management	Outcome
1	Yahchouchy-Chouillard et al. (2002) (5)	73/M	Indirect inguinal	Right	TC with diverticulitis, ileal loops, omentum	TC diverticulitis simulating strangulation	Large, long-standing hernia with wide neck; diverticulitis secondary to incarceration	Shouldice repair; conservative treatment of diverticulitis	Uneventful recovery
2	Tan et al. (2007) (6)	69/M	Indirect inguinal	Left	TC with synchronous carcinoma, omentum	Bowel obstruction; synchronous ascending colon carcinoma	Mechanical propulsion by tumor mass aided by gravity	Extended right hemicolectomy, primary anastomosis, hernia repair	Slow but good recovery; discharged POD 37
3	Tiwary et al. (2008) (7)	55/M	Indirect inguinal	Right	Gangrenous TC with omentum (part of massive bowel gangrene: terminal ileum to proximal TC)	Massive bowel gangrene due to SMA thrombosis; septic shock	Superior mesenteric artery thrombosis (not hernia strangulation)	Extended right hemicolectomy, end ileostomy, hernia repair	Survived; discharged POD 18; well at 2-month follow-up
4	Singal et al. (2012) (8)	64/M	Indirect inguinal	Right	Gangrenous ascending colon and proximal TC, appendix tip	Gangrenous bowel, acute appendicitis	Mobile cecum; long-standing hernia	Right hemicolectomy, ileotransverse anastomosis, orchiectomy, hernia repair without mesh	Asymptomatic at 4-month follow-up
5	Lee (2012) (9)	75/M	Indirect inguinal (giant)	Right	Proximal TC to terminal ileum with mesentery (malrotated intestine)	None (no ischemia or strangulation)	Intestinal malrotation with incomplete fixation; loss of domain	Ladd's procedure, appendectomy, inguinal defect closure	Uneventful; discharged POD 3
6	Burke et al. (2014) (10)	89/F	Inguinal (bilateral; left irreducible)	Left (bilateral)	TC loop with saccular metastatic deposits (ovarian carcinoma; imaging-based; no surgical confirmation)	Disseminated ovarian carcinoma, omental disease, ascites	Increased intra-abdominal pressure from ovarian malignancy and ascites	Conservative (palliative); not a surgical candidate	Died 6 weeks after discharge
7	Dinesh et al. (2014) (11)	45/M	Indirect inguinal (giant)	Left	Omentum, TC with parts of ascending and descending colon	None (no strangulation)	Giant, long-standing (25-year) hernia with loss of domain	Lichtenstein mesh repair, partial omentectomy	Uneventful; mild scrotal hematoma resolved by 3 months
8	Ota et al. (2015) (12)	79/M	Inguinal (type not specified)	Left	TC (imaging-based; no surgical confirmation)	TC perforation into scrotum; panperitonitis	History of prior gastrectomy (possible altered anatomy); large hernia defect	Patient refused surgery; conservative (antibiotics)	Died of panperitonitis 19 days after admission
9	Diao & Ghosh (2016) (13)	48/M	Indirect inguinal	Left	TC with perforated carcinoma	TC carcinoma perforated into scrotum; scrotal abscess	Tumor mass acting as lead point for herniation	Transverse colectomy via inguinoscrotal incision, primary anastomosis, hernia repair without mesh; subsequent orchiectomy/scrotectomy	Uneventful; no recurrence at 1 year
10	Aldhafar et al. (2020) (14)	53/M	Indirect inguinal	Right	TC, greater omentum	Omental ischemia	Not explicitly discussed; large hernia defect	Omentectomy, mesh repair (Lichtenstein)	Uneventful recovery; discharged POD 7
11	Bayissa & Borena (2023) (15)	65/F	Femoral	Right	TC, omentum	Incarceration (no strangulation)	Postmenopausal factors, chronic constipation, wide femoral ring	Open femoral herniorrhaphy, partial omentectomy	Uneventful; no recurrence at 4 months
12	Alnumay et al. (2025) (16)	67/M	Indirect inguinal	Right	TC, omentum	Closed-loop bowel obstruction	Long-standing (20-year) hernia with progressive enlargement	Modified Shouldice repair with mesh	Uneventful; discharged POD 4; well at 6-month follow-up
13	Czyzewski et al. (2025) (17)	63/M	Indirect inguinal (giant; 39 years)	Right	Right colon, TC, greater omentum	Scrotal hematoma (postoperative)	Giant, long-standing (39-year) hernia with loss of domain	Omentum resection, Lichtenstein mesh repair	Recovered after hematoma drainage
14	Present case	58/M	Indirect inguinal	Right	TC only	Strangulation with TC necrosis; cecal perforation	Mesenteric shortening secondary to sclerosing mesenteritis causing inferior displacement of TC	Right hemicolectomy, protective loop ileostomy, Bassini repair; subsequent stoma closure	Uneventful; successful stoma closure at 3 months

TC, transverse colon; SMA, superior mesenteric artery.

A sliding hernia is a type of inguinal hernia in which a viscus and its overlying peritoneum form part of the hernia sac wall as the organ protrudes into the inguinal canal; the sigmoid colon, cecum, and urinary bladder are the most commonly involved organs (19). Its development is related to the degree of posterior fixation of the involved organ and its proximity to the internal inguinal ring (4). In contrast, the transverse colon is entirely intraperitoneal and suspended by the transverse mesocolon, which ordinarily restricts its inferior displacement. Consequently, the mechanism underlying transverse colon herniation is fundamentally different from that of a sliding hernia and cannot be explained by the same anatomical principles. This distinction necessitates the identification of alternative predisposing factors in each reported case.

Review of the published cases reveals several distinct mechanisms that may predispose the transverse colon to groin herniation. The most frequently observed pattern is concurrent omental herniation, in which the omentum appears to act as a lead point that facilitates migration of the transverse colon into the inguinal canal via a traction effect. This pattern has been described in both inguinal (14, 16) and femoral hernias (15), the latter representing the only reported case of transverse colon herniation through the femoral canal. Giant or long-standing inguinal hernias with loss of abdominal domain represent another predisposing condition. Several reports have documented transverse colon herniation in the setting of giant hernias present for decades, often accompanied by concurrent herniation of other colonic segments and omentum, with chronic enlargement of the hernia defect and progressive loss of domain as the primary predisposing factors (9, 11, 17). Malignancy has also been implicated in transverse colon herniation, with reported cases involving primary transverse colon carcinoma acting as a propulsive lead point (6, 13) and metastatic ovarian carcinoma with saccular deposits within the hernia sac (10). These cases highlight the role of tumor-related mass effect and increased intra-abdominal pressure in facilitating transverse colon displacement. Other reported mechanisms include superior mesenteric artery thrombosis with massive bowel gangrene incidentally involving the transverse colon (7), a mobile cecum facilitating herniation of the ascending colon and proximal transverse colon (8), and transverse colon diverticulitis simulating hernia strangulation (5). Notably, among all reported cases, isolated transverse colon herniation—without concurrent omental, small bowel, or other visceral involvement—was documented only in the present case. This observation underscores the rarity of isolated transverse colon displacement and suggests that a specific predisposing factor, rather than a large hernia defect or omental traction alone, is required for the transverse colon to herniate independently.

SM is a rare, benign fibroinflammatory disorder of the mesentery that encompasses a histopathological spectrum ranging from predominantly inflammatory forms (mesenteric panniculitis) to fibrosis-dominant forms (retractile mesenteritis), now accepted as a single disease continuum under the unifying term of SM (20). The condition predominantly involves the small bowel mesentery and occurs most often in men during the fifth to seventh decades of life (20). Critically, SM should not be regarded merely as a radiological curiosity: in a series of 92 patients, Akram et al. reported that complications included small bowel obstruction in 24% and mesenteric venous thrombosis in a minority, reflecting the potentially serious mechanical consequences of progressive mesenteric fibrosis (21). The advanced fibrotic stage—retractile mesenteritis—produces dense collagen deposition and desmoplastic retraction that can physically shorten, tether, and distort the mesentery and its attached bowel loops (20, 22). Although the condition most commonly affects the small bowel mesentery, colonic mesentery involvement has also been reported, with Williams and Nelson describing two cases of retractile mesenteritis presenting with colonic obstruction as the dominant clinical feature (23).

**Figure 2:**
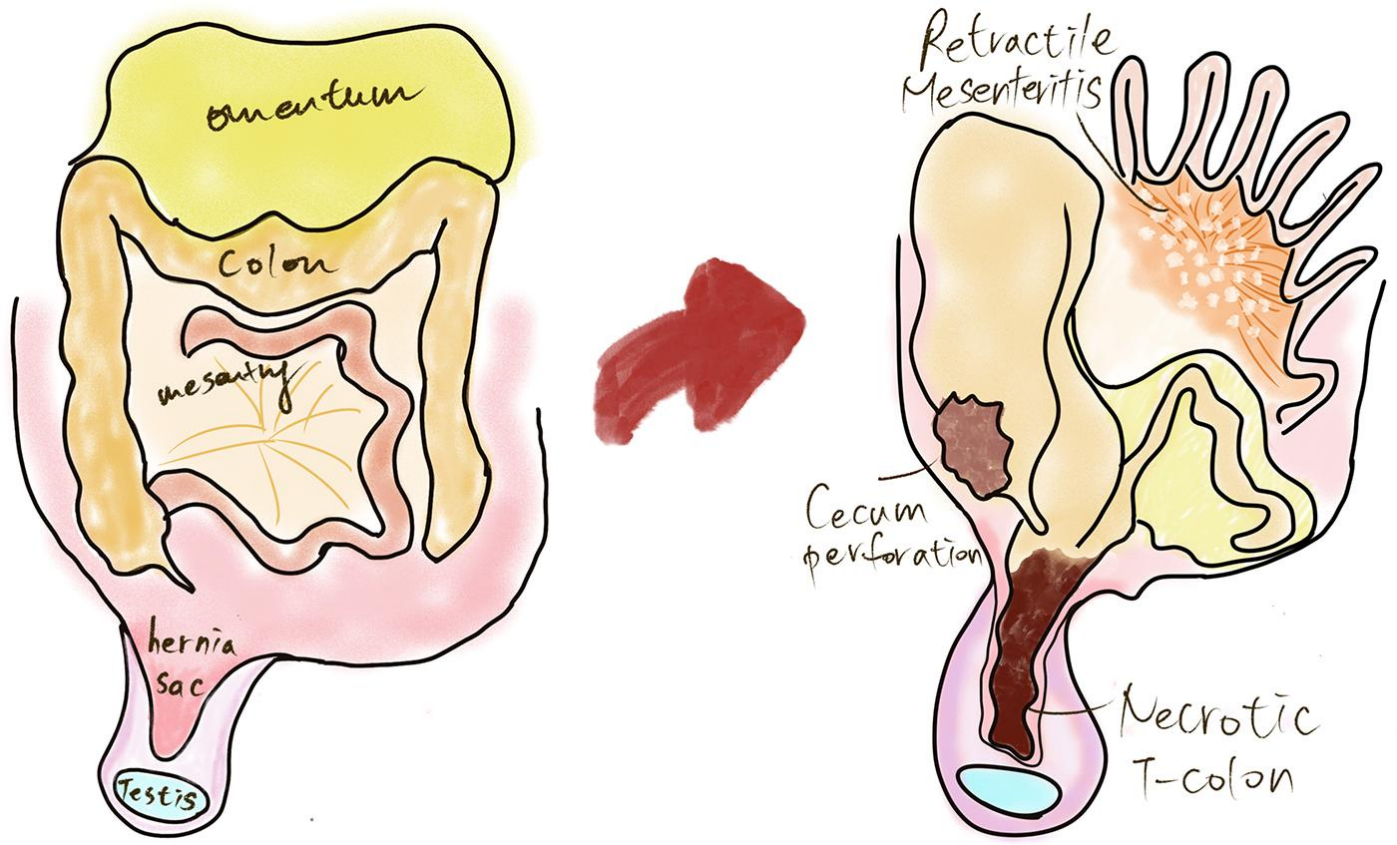
A schematic illustration of the proposed mechanism of transverse colon herniation. Mesenteric fibrosis and shortening may have displaced the transverse colon inferiorly into a pre-existing right indirect inguinal hernia, while the omentum remained intra-abdominal.

We propose that transverse colon herniation in our case resulted from the convergence of multiple predisposing factors (Figure 2). First, progressive fibrosis within the small bowel mesentery produced desmoplastic retraction and physical shortening, drawing the small-bowel loops superiorly toward the mesenteric root. This clustering of the small bowel created a relative vacancy in the lower abdominal cavity and eliminated the normal mechanical buffering that keeps the transverse colon in its supra-umbilical position. Second, the patient had a pre-existing chronic right inguinal hernia that had been present for several months and was previously reducible, providing a persistent fascial defect through which displaced bowel could herniate. Third, it is plausible that a degree of inherent transverse colon redundancy facilitated its descent to the level of the inguinal canal once spatial constraints were removed. The interplay of these factors—mesenteric shortening displacing the transverse colon inferiorly, a chronic hernia defect providing the exit pathway, and possible colonic redundancy permitting sufficient mobility—offers a more plausible mechanistic explanation than any single factor alone. Notably, no omental co-herniation was present, distinguishing this case from the majority of previously reported cases and suggesting that inferior displacement of the transverse colon was driven by the loss of normal intra-abdominal spatial constraints rather than by traction from co-herniating structures.

Although a direct causal link cannot be proven, this hypothesis is supported by converging lines of evidence. SM has been documented to cause small bowel obstruction and volvulus through mesenteric foreshortening (24). Furthermore, other fibroproliferative mesenteric lesions, such as mesenteric fibromatosis, have been reported to cause transverse colonic volvulus, demonstrating that mesenteric fibrosis can specifically alter transverse colonic anatomy (25). To our knowledge, this is the first report to link SM to transverse colon displacement and subsequent inguinal herniation.

The surgical strategy in this case was dictated by the dual challenges of bowel necrosis with perforation and peritoneal contamination. While tension-free mesh repair is the standard of care for elective inguinal hernia repair, its use in contaminated fields is associated with high risks of infection and mesh-related complications. In such circumstances, tissue-based repair remains the preferred option (26). In our patient, right hemicolectomy was required for the non-viable colon, and a protective loop ileostomy was created to mitigate the risk of anastomotic leakage. Given the degree of contamination, mesh was contraindicated, and a Bassini repair was performed, providing reinforcement of the posterior wall while minimizing the risk of prosthetic infection.

## Strengths and limitations

A major strength of this case is the correlation between the operative findings and the histopathological features of fibrosis-dominant SM. However, as a single case report, this observation remains insufficient to establish a definitive causal relationship between SM and transverse colon herniation, and direct literature support for this mechanism remains limited.

## Conclusions

In summary, ours is the first reported case of transverse colon herniation occurring without concurrent omental or visceral involvement, specifically within the context of SM with mesenteric shortening. This potential pathological association suggests that mesenteric fibroinflammatory disease may warrant consideration in the differential diagnosis of atypical hernia contents. In emergency settings complicated by ischemia and contamination, bowel resection with fecal diversion and tissue-based hernia repair remain the standard of care. Further investigation is warranted to clarify the role of mesenteric disease in such rare clinical presentations.

## Data Availability

The original contributions presented in the study are included in the article/Supplementary Material, further inquiries can be directed to the corresponding author.
